# Routine Change of Nasogastric Tube in Intensive Care Unit: Friend or Foe

**Published:** 2011-05-01

**Authors:** F Zand, G Sabetian, Z Ghodrati

**Affiliations:** 1 Department of Anesthesiology, Anesthesiology and Critical Care Medicine Research Center, Shiraz University of Medical Sciences, Shiraz, Iran

**Keywords:** Change, Nasogastric tube, Intensive care unit

Dear Editor,

Early enteral feeding is a standard of care in many intensive care units (ICU) around the world. The use of enteral route is facilitated by the development of highly flexible nasogastric tubes (NGT). Although the insertion of nasogastric tubes has been described as being easy, but can produce unexpected complications. The insertion of a feeding tube into the lower airway is an uncommon complication which is more likely to occur in obtunded or intubated patients.[1]

We describe a near fatal complication due to NGT malposition in an intubated patient, after routine change of nasogastric tube. The object of this case presentation is to mention three points including i) To question the need for routine change of NGT and its complications, ii) To show the possible increased risk of delayed detection of NGT malposition in the airways during continuous nasogastric feeding and iii) To bring up the possible role of early bronchoscopy and bronchial lavage for treatment of massive bronchial aspiration of food material.

Our case was a 38 year male case of Guillain-Barre syndrome. The patient was intubated in third day of his admission due to progressive muscle weakness and respiratory distress. Entral nutrition via a nasogastric tube was begun on the forth day of ICU admission. Continuous NG feeding of 20 ml/hr was started and reached to 80 ml/hr during a 3 days period. The patient was conscious, orient and under mechanical ventilatory support. The mode of ventilator was pressure support of 10 cmH2O with inspiratory oxygen concentration (FiO2) of 40%, PaO2/FiO2=300. NGT was changed according to an institutional protocol on 4th day and its location was confirmed by epigastric auscultation (whoosh test) as routine without any aspiration, then continuous feeding was restarted.

**Fig. 1:**
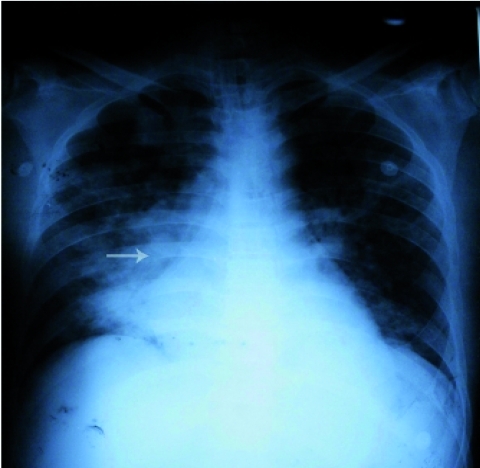
Aspirated material through malpositioned nasogastric tube in right main bronchus

After about 1 hour, the patient's respiratory status deteriorated, O2 saturation decreased to 85%, requiring increase of FiO2 to 100% and to change mode of ventilator to synchronize the intermittent mandatory ventilation. At this point, the PaO2/FiO2 was 80. XRay film after 4 hours showed the NGT to be in right main bronchus and pulmonary infiltration in right middle lobe. The NGT was replaced followed by aspiration of grossly visible food material, and positive whoosh test over the epigastrium after rapid injection of 40 ml of air. Second chest x-ray revealed the feeding tube to be in the right main bronchus (Figure 1). Feeding tube was removed and emergency bronchoscopy was done. Because of aspiration of large amount of feeding material (about 400 ml), we irrigated the tracheobronchial tree with 600 ml normal saline and broad spectrum antibiotics (imipenem, vancomycin and amikacin). After 5 hours, the patient's condition was better. PaO2/FiO2 increased to 200, mode of ventilator was changed to SIMV with FIO2 40% and after 24 hours to pressure support.The white blood cell count decreased from 13200 to 5800/ml after 6 days, procalcitonin level decreased gradually after 5 days (first day>10, 5th day> 0.5 and 9th day< 0.5 unit). Sputum culture was positive for Klebsiella species.

We reviewed the literature and there were few recommendations about time of NGT change in ICU. Insertion of feeding tube may bear some complications. The incidence of misplacement of NGT has been reported to be 0.3-27%.[2][3] Pleural effusion, lung abscess, retropharyngeal abscess, pneumonitis,[4][5] erosion into major vessel,[6] direct vascular placement,[7] following NGT insertion have all been reported. The intragastric position of the tube must be confirmed after initial placements before each feeding. A few confirmatory tests have been recommended for detection of NGT misplacement while none of them has been considered to be perfect.

Clinical signs were easy placement of the tube to it's full length, the absence of coughing, visual inspection of gastric juice aspiration and positive epigastric auscultation.[8] These methods are useful when obvious, but can be extremely misleading and are all unreliable.[8] Benya found a 20% false gastric confirmation by auscultation.[9] Duthorn recommended an initial negative blood aspiration test before air insufflation, because of possibility of fatal complication of direct vascular placement of tube.[7] A chest roentgenogram was the gold standard for confirming the position of enteral tube,[4] but misinterpretation of the radiograph can occur specially in complex critically ill patients. Other paraclinical tests such as pH of aspirate and bilirubin helped the confirmation.[3] Capnography compromised the presence of carbon dioxide (CO2) as a proven surrogate marker for the pulmonary environment.[8]

It is important to emphasize that tracheal misplacement of a NGT may be clinically silent and its diagnosis needs a high index of suspicious, because of its outcome.

The presence of an endotracheal tube or tracheostomy does not provide protection against tracheobronchial misplacement. There may be a false positive result in common confirmatory tests.
